# Placental–Heart Axis: An Evolutionary Perspective

**DOI:** 10.3390/ijms252011212

**Published:** 2024-10-18

**Authors:** Jadyn Matthews, Brammy Rajakumar, Chrystalle Katte Carreon, Sarah U. Morton

**Affiliations:** 1 Division of Newborn Medicine, Department of Pediatrics, Boston Children’s Hospital, Boston, MA 02115, USA; jadynmatthews@college.harvard.edu (J.M.); brajakumar@hms.harvard.edu (B.R.); 2 Department of Pathology, Boston Children’s Hospital, Boston, MA 02115, USA; katte.carreon@childrens.harvard.edu; 3 Department of Pathology, Harvard Medical School, Boston, MA 02115, USA; 4 Department of Pediatrics, Harvard Medical School, Boston, MA 02115, USA

**Keywords:** placenta, evolutionary biology, congenital heart disease

## Abstract

To maintain its development, the growing fetus is directly dependent on the placenta, an organ that acts as both a modulator and mediator. As an essential component of pregnancy that is derived from both maternal and fetal tissues, the placenta facilitates the passage of all oxygen and nutrients from the expecting parent to their fetuses. Further, the placenta conveys multiple impacts of the maternal environment to the growing fetus. The timing of placental development parallels that of the fetal cardiovascular system, and placental anomalies are implicated as a potential cause of congenital heart disease. For example, congenital heart disease is more common in pregnancies complicated by maternal preeclampsia, a condition characterized by placental dysfunction. Given the placenta’s intermediary links to the maternal environment and fetal health outcomes, it is an emerging focus of evolutionary medicine, which seeks to understand how interactions between humans and the environment affect our biology and give rise to disease. The present review provides an overview of the evolutionary and developmental courses of the placenta as well as their implications on infant health.

## 1. Introduction

**Figure 1:**
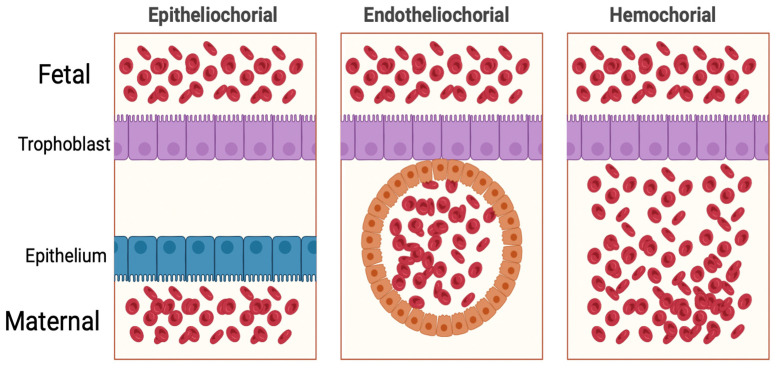
Placental classifications. Created using BioRender.

**Figure 2:**
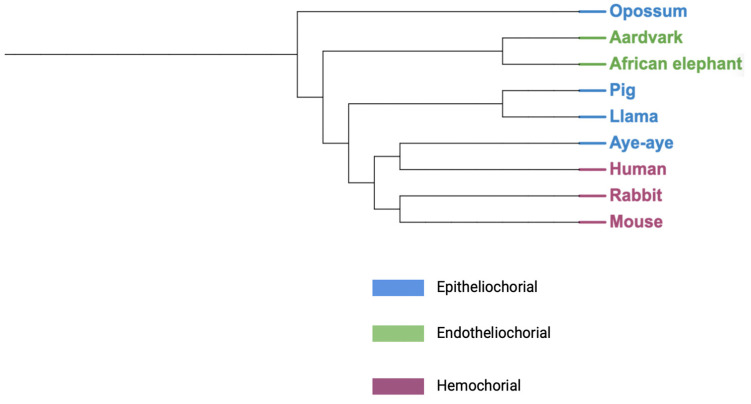
Placental classifications among mammals. Created using BioRender.

The placenta is an essential organ during human gestation, and its impact on human health can be trans-generational. In 1937, Mossman offered a universal definition of the mammalian placenta: “fusion of the fetal membranes to the uterine mucosa for physiological exchange” [1]. While not comprehensive, this definition is helpful in evolutionary and comparative biology contexts because it captures the bidirectional role of the placenta across species. Through convergent evolution, placentas have emerged in all vertebrates, apart from birds [2]. Even stingrays exhibit finger-like protrusions from the uterus to transfer nutrients to the embryo during gestation. Across species, there are three main classes of placentas: epitheliochorial, endotheliochorial, and hemochorial (Figure 1). These classes differ significantly in the amount of separation between the placental trophoblast layer and maternal vasculature. Epitheliochorial placentas do not erode the uterine epithelium, and therefore have a separation of the trophoblastic layer and the maternal epithelium. Endotheliochorial placentas have contact between the trophoblasts and the maternal vascular epithelium without penetrating maternal blood vessels. Finally, hemochorial placentas have trophoblast layers that implant into the uterine epithelium and directly border maternal capillaries. Phylogeny and convergent evolution point to the placenta of the last common placental mammal ancestor having a discoid hemochorial placenta, like those of humans and mice, or one that was discoid and endotheliochorial, such as the aardvark and African elephant (Figure 2) [2,3]. As such, the last common ancestor of hominids is presumed to have a hemochorial placenta which will share many characteristics with model organism species such as the mouse. However, there are also important divergences between human and model organisms such as mouse and rabbit, highlighting the need to understand human placental biology and pathology. Understanding the evolutionary relationship between placental mammals can also highlight potential mechanisms of development and disease. A deeper understanding of these mechanisms can enable new therapeutic modalities to the benefit of the expecting parent and their fetuses.

## 2. Human Placental Development

In humans, the placenta begins to form during the first week after fertilization once blastocyst implantation is complete [4,5]. At approximately 12 days gestation, the maternal circulation to the developing placenta is established [6]. Maturation continues over the first trimester and beyond [4,7]. Human placentas have trophoblast origins, a feature unique to mammals [2]. Comparatively, the embryonic degradation of trophoblast cells is considered the initial lineage determinant of Mammalia. Once the blastocyst implants into the uterine endometrium, the trophoblast differentiates into the syncytiotrophoblast and cytotrophoblast [7]. The syncytiotrophoblast secretes human chorionic gonadotrophic hormone, signaling implantation and pregnancy, and the cytotrophoblast secretes enzymes to breakdown portions of the uterine endometrial wall which enables placental invasion [4,8].

**Figure 3:**
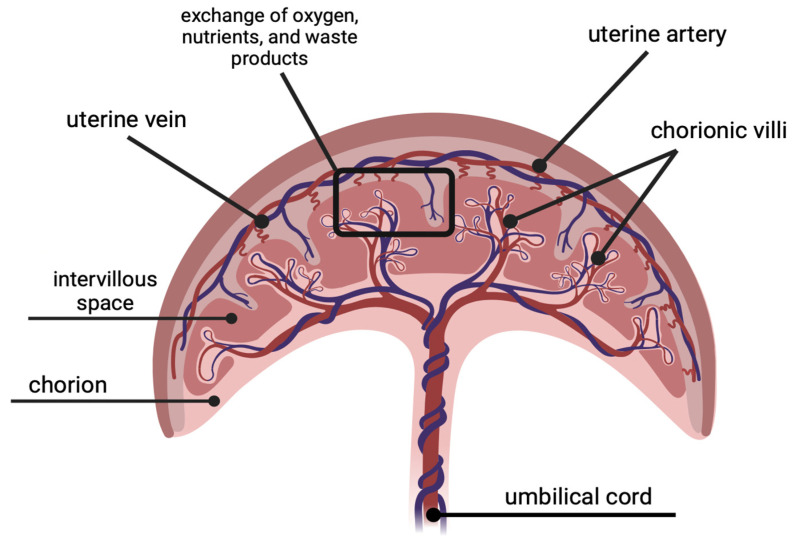
Placental circulatory system. Created using BioRender.

The placenta, placental membrane, umbilical cord, and amniotic fluid are all considered placental tissues [9]. Human placental tissues are derived from both maternal and fetal cells to produce distinct uteroplacental and fetoplacental circulatory systems [9,10,11]. The placental membrane consists of three layers: the amnion, the chorion, and an intermediary collagen and proteoglycan network [9]. The amnion layer is fetal facing and acts to protect and enclose the growing fetus in amniotic fluid [9,10]. The chorion, which includes the syncytiotrophoblast and cytotrophoblast, is maternal facing [7,9]. The chorionic villi, which protrude into the intervillous space and enable oxygen and nutrient transfer across the placental membrane, are created when the cytotrophoblast encroaches into the syncytiotrophoblast (Figure 3). Further growth of the villi allows for the emergence of blood vessels and capillary beds. A key structure that these capillaries connect to is the embryo’s heart, the first organ to develop during organogenesis [7,12]. This network provides the basis for fetal circulation from the two umbilical arteries to the placenta, then returns with increased oxygen via the umbilical vein. The placenta is the largest organ for a growing fetus and receives almost half of fetal cardiac output [13]. While the amniotic and chorionic layers are histologically separate, the fetal and maternal vessels are still able to exchange nutrients, gases, and waste products in the placenta through passive and active transport [7,9,10]. Thus, all these tissues are essential to placental function.

**Figure 4:**
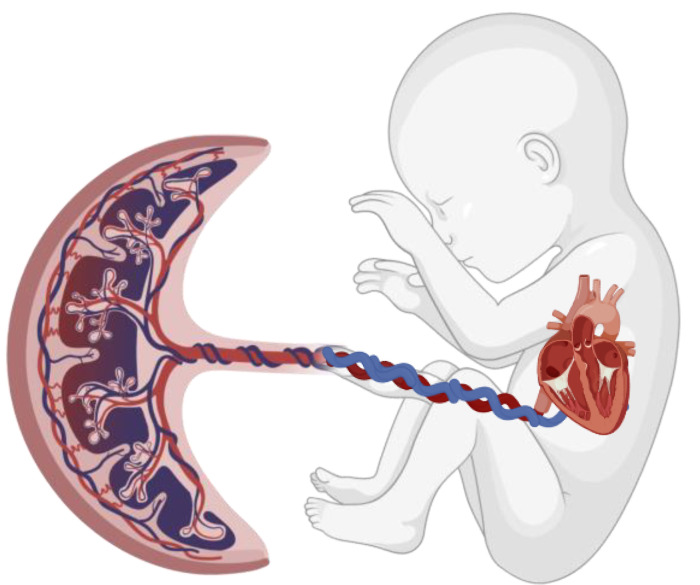
Placental–fetal circulation. Created using BioRender.

Placental implantation in humans is considered highly invasive as the embryonic trophoblast fully embeds into the uterine endometrium [1,14]. Invasion into the uterine endometrium may facilitate gas and nutrient transport, particularly of macromolecules but could also transmit non-beneficial factors such as toxins [2,15]. The efficient and successful facilitation of oxygen by the placenta during gestation is an adaptive process that likely resulted from natural selection in light of changing environmental oxygen levels over evolutionary time [1]. The placental circulation provides oxygenated blood to the fetus which enters the right atrium, and then returns to the placenta after exiting the left ventricle (Figure 4). The first three months are also when fetal organ development occurs and mark a time when the fetal microenvironment is particularly oxygen scarce. The placenta is also well-adapted to serve as a physiological defense from outside pathogens to the vulnerable fetus [1]. However, aspects of modern culture, including pollution and smoking, inhibit the placenta’s protectiveness from the maternal environment. For instance, pregnant women living in urban-industrial areas have shown increased levels of environmental pollutants, lead, and cadmium, in their placentas compared to those living in more rural environments [15]. The placenta therefore acts as an important mediator of the maternal environment impacts on the developing fetus, including growth and risk of congenital malformations [15,16].

## 3. Placental Dysfunction

Placental function and dysfunction are key modulators of fetal development and have long-lasting implications for child health [17]. For instance, maternal hypertensive disorders such as preeclampsia have been linked to increased risk of fetal congenital heart disease [18]. Female infants born to those reporting more depression symptoms have reduced placental telomere length [19]. Placental dysfunction has also been implicated in impaired neurodevelopment and brain growth [20]. To standardize the assessment of placental pathology across clinical care and research, the Amsterdam Placental Workshop Group established a consensus for diagnosing placental pathology [21]. Four prevalent and significant types of placental injury were identified: maternal vascular malperfusion, fetal vascular malperfusion, acute chorioamnionitis, and villitis of unknown etiology. These patterns can present with wide-ranging effects on the placenta [22]. For example, maternal vascular malperfusion can be caused by alterations in differentiation signals that prevent the remodeling of the spiral arteries and result in obstructed blood flow, and this has implications for the placenta’s growth [22]. As such, maternal vascular malperfusion is found in pregnancies with severe maternal preeclampsia, a condition characterized by high maternal blood pressure and associated with significant risks to the mother and fetus. Maternal vascular malperfusion has been associated with fetal growth restriction, preterm birth, placental abruption, and increased risk for future cardiovascular disease in both the parent and child [22]. Severe maternal vascular malperfusion in preterm babies is also associated with poor neurological outcomes, including cerebral palsy, epilepsy, and cognitive impairment [17]. Maternal diabetes, linked to placental anomalies such as fetal vascular malperfusion, is associated with congenital heart disease in mice and humans [22,23,24]. As a potential mediator for many maternal and environmental risk factors known to contribute to risk for congenital heart disease, advances in our understanding of placental disorders could also improve our knowledge about the origins of congenital malformations.

## 4. Congenital Heart Disease

Congenital heart disease is the most prevalent severe congenital anomaly in humans [25]. Approximately 1% of annual births in the United States are infants affected by congenital heart disease, a quarter of which are critical and therefore require surgical intervention during infancy [26]. Advances in medical and surgical care have led to improvements in many outcomes: between 1979 and 1993, about 67% of infants with critical congenital heart disease survived to one year, and this number rose to about 83% between 1994 and 2005 [27]. Despite advances in medical and surgical care, congenital heart disease is a significant contributor to infant mortality, comprising almost 5% of all neonatal deaths in the United States [26]. Research efforts aiming to fully elucidate the direct causes of congenital heart disease continue, with a growing body of evidence pointing to the potential role of genetic risk to the majority of congenital heart disease [28]. It is also known that placental anomalies are more frequent in pregnancies with fetal congenital heart disease, and multiple subtypes of congenital heart defects, including Tetralogy of Fallot, have been found to be associated with lower placental weight [4,29]. Therefore, an improved understanding of placental biology and dysfunction has emerged as a promising path towards uncovering additional risk factors for congenital heart disease [17].

Neurodevelopmental impairment is the most common morbidity and occurs in up to 50% of children with moderate to severe congenital heart disease [30]. Several patient and pre-operative factors are known to have a higher impact on later neurodevelopmental risk than later postoperative factors, particularly in predicting neurodevelopmental outcomes. There are currently two main innate factors for neurodevelopmental disabilities associated with congenital heart disease: prenatal hemodynamic and genetic variants. First, the developing brain is dependent on fetal cardiac and placental function for delivery of oxygen and nutrients [31]. Altered hemodynamics associated with congenital heart disease results in abnormal brain structure in fetuses and neonates with congenital heart disease, and reduces proliferation and neurogenesis in the subventricular zone, which is accompanied by reduced cortical growth [32]. Second, recent studies have demonstrated shared genetic regulation of heart and placental development [33]. Genetic variants could lead to increased resiliency or increased risk to brain development depending on their impact. However, the extent to which these two factors impact neurodevelopment among people with congenital heart disease, and which factors are the best targets for interventions, remains an active area of research.

## 5. Placental–Heart Axis

Placental pathologies have been identified in 57% of pregnancies with fetal congenital heart disease [34]. Specifically, a high prevalence of maternal vascular malperfusion and fetal vascular malperfusion has been found in multiple case–control studies [17]. These pathologies indicate obstruction of maternal and fetal blood flow to the placenta, respectively. A chronic inflammatory maternal T-cell-mediated placental lesion known as villitis of unknown etiology has also been found in approximately 10% of placentas from pregnancies with fetal congenital heart disease. This is remarkable because high-grade villitis of unknown etiology has been previously associated with neurological impairment and cerebral palsy, thought to be secondary to the obliterative vasculopathy caused by the inflammation, which results in loss of functional downstream chorionic villi, analogous to primary fetal vascular malperfusion. More research is needed to understand the mechanism of these placental changes, and ultimately to identify potential therapeutic targets that could remedy placental dysfunction.

Given humans and mice share a discoid placenta with hemochorial formation, experimental mouse models can shed light on placental and cardiac conditions that have yet to be causally linked in humans [2]. For example, mouse models can be used to characterize the role of gene expression in the developing placenta and heart. Some genes such as *Asb2*, which facilitates actin remodeling by degrading filamins, are required for both placental and fetal cardiac development [12]. Disturbance of the *Vcam1* gene, which encodes vascular cell adhesion molecule 1, hinders placentation and leads to fetal cardiovascular anomalies [23,35]. Low placental VCAM1 is also associated with constrained growth trajectories in human infants [23]. In a tissue-specific genetic model, loss of trophoblast expression of *Atp11a*, a phospholipid flippase gene present in the heart and placental syncytiotrophoblast, leads to septal defects and abnormal ventricular development despite preserved embryonic expression [33]. In contrast, loss of embryonic expression of *Ssr2* causes septal defects and abnormal ventricular development, while loss of *Ssr2* expression in trophoblasts did not result in significant changes to cardiac development. Thus, placental expression of *Atp11a* and cardiac expression of *Ssr2* are necessary for typical cardiac development. *Smg9*, a gene involved in mRNA regulation and decay, has been linked to both preeclampsia and congenital heart disease [33]. Recent investigation discovered the role of endogenous progesterone immunomodulatory binding factor 1 (PIBF1) in trophoblast differentiation and fusion into syncytiotrophoblast in humans and mice [36]. PIBF1 facilitates cellular crosstalk between syncytiotrophoblast and adjacent vascular cells, which is crucial for the establishment of vascular network in the developing [36]. In mice, this process has also been shown to influence early development of the embryonic cardiovascular system. Additionally, in vitro experiments showed that PIBF1 facilitates development of cardiovascular characteristics in heart organoids [36]. These mouse studies have provided strong support for a placenta-heart axis, emphasizing the possible interdependence of these two vital organs, during development.

## 6. Placental–Heart–Brain Axis

Along with the increasing recognition that maternal placental malperfusion is common in pregnancies affected by fetal congenital heart disease, an emerging field of neuroplacentology has identified an association between placental dysfunction and later risk for long term neurodevelopmental delays in people with congenital heart disease. Recent studies have implicated placental health in neurodevelopmental outcomes among people with congenital heart disease. Brain magnetic resonance imaging studies have offered insight about neurodevelopmental impairment, and there is now considerable evidence that abnormal brain volume, white matter structure, and gyrification occur in congenital heart disease before neonatal cardiac surgery and are present even before birth [37,38,39,40,41,42,43,44,45,46]. Pre-operative white matter injury has been observed from brain magnetic resonance imaging obtained in neonates with congenital heart disease, highlighting the likelihood of prenatal brain injury related to congenital heart disease [20]. Up to 23% of newborns with congenital heart disease were found to have a brain injury pre-operatively [47,48]. These injuries can be accompanied by reduced frontal lobe and brainstem volumes [20,41]. Several types of congenital heart disease, including common arterial trunk, single ventricular defects, and anomalous pulmonary venous return, are associated with smaller head circumference—a commonly used infant measurement in clinical practice [29]. Importantly, a higher incidence of severe brain injuries was found in infants with congenital heart disease who also had abnormal placental pathology [34]. This increased incidence of brain injury for patients with congenital heart disease, especially after cardiac surgery, translates to further increased risk for negative neurological outcomes at discharge, including cognitive, language, and motor delays and regulation difficulties related to state management, feeding, and sleeping [49]. These risks are compounded by the presence of neurological events occurring during pre-, peri-, or post-operative periods for these patients [49]. As people with congenital heart disease experience excess risk of longer term cognitive, motor, language, and emotional/behavioral problems, these placenta pathologies could be an important mediator between the maternal environment, fetal heart, and the fetal brain [17,50].

It is hypothesized that improving placental function may improve fetal well-being including overall growth and brain development. Given that fetal hypoxia may be one important mechanism of prenatal neurodevelopmental risk, ongoing clinical trials are assessing the role of maternal hyperoxygenation via noninvasive nasal cannula in pregnancies affected by single ventricle congenital heart disease, a particularly severe form of congenital heart disease [51]. It is believed that maternal hyperoxygenation therapy leads to an increase in fetal pulmonary blood flow as well as increased heart growth in fetuses [52]. Other interventions aimed at nutrient modification or improvement of fetal cardiac function could also modify the relationship between placental dysfunction, fetal cardiac status, and fetal brain development.

## 7. Evolutionary Medicine

In addition to its biological significance, the placenta is a unique keystone organ due to its mediatory relationship between the fetus and the parent [16]. Here, it serves as a conduit to provide important markers from the broader maternal environment to the growing fetus. The field of evolutionary medicine seeks to understand how interactions between humans and their environment both propagate and prevent disease [53,54]. For example, evolutionary perspectives aid in explanations of heterozygote advantage, such as with individuals carrying the sickle cell allele, *HbAS*, having resiliency during malaria infection [53]. Areas where malaria is endemic, particularly in Sub-Saharan Africa, have seen high persistence of *HbAS* heterozygosity in their populations, enabling increased protection against malaria while avoiding sickle cell disease, which arises in individuals homozygous for *HbAS*.

Evolutionary perspectives also identify numerous health conditions that result from mismatches between humans and their environments [53]. Type 2 diabetes, a dramatically increasing condition projected to double in prevalence by 2050, can be analyzed through the mismatch hypothesis [55,56]. Evolutionary theory points to humans’ ancestors being skilled hunter–gatherers who were well-adapted to their environments [56]. Adaptiveness to this lifestyle spanned locomotive and metabolic means, including a high capacity for physical activity through distance hunting and a proclivity for high-energy foods, such as meat and grains [56,57,58]. As humans transitioned from hunter–gatherer lifestyles to subsistence agriculture, and, most recently, industrialized society, they created heavily altered environments that do not resemble our ancestors’ [56,59]. For many people, modern life is more sedentary and includes a high degree of processed and sugary foods, representing an environment and diet for which humans’ bodies are not adapted and a pathway for metabolic dysfunction diseases, like diabetes, to emerge [56,60,61].

**Figure 5:**
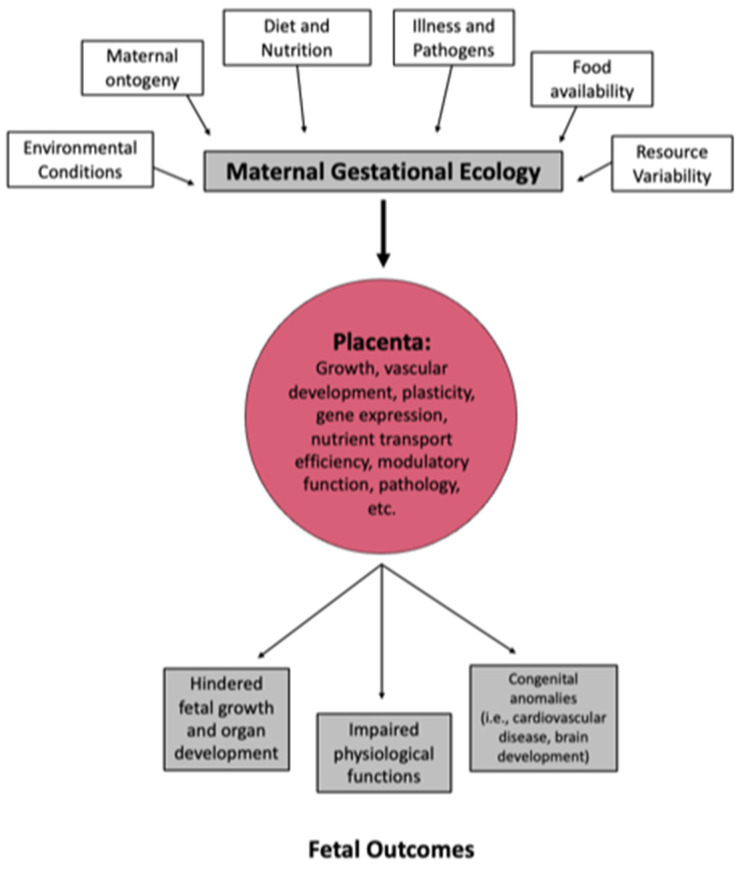
Multiple components of maternal ecology can contribute to changes in placental function, which in term mediate fetal outcomes.

Modern behaviors can be a facilitator of numerous diseases, and evolutionary medicine explains how sickness can arise when a population lives in “an environment that exceeds its adaptive capacities” [61]. When broadening the scope of the environment from purely climate considerations to an individual’s social situation and surroundings, one arrives at the basis for health care’s increased focus on social determinants of health over the past 30 years [62]. Social determinants of health broadly encompass social factors in one’s surroundings that influence health outcomes [63]. These social drivers include food availability, employment, pollution exposure, housing safety, socioeconomic status, health care and insurance accessibility, and education level, and they are thought to impact the functions of multiple physiological processes, including the autonomic, cardiovascular, and metabolic systems [62,63,64]. This additional life course lens furthers considerations into how cultural changes to the environment influence human health and susceptibility to disease [58,63]. When considering how these environmental factors could affect a growing fetus, Rutherford [65] intelligibly offers a portrayal of the relationship that not only applies to humans but spans species: “the intrauterine environment encountered by the fetus is largely a function of maternal ecology: the nexus of nutritional, metabolic, endocrinological, infectious, genetic, epigenetic, and socio-behavioral inputs that coalesce into a particular pregnancy” (Figure 5). Potential factors that can alter the maternal gestational ecology include all of the aforementioned domains and are important to consider as potentially modifiable factors that can increase resilience when encountering adversity or risk for adverse outcomes. The physical manifestation of these impacts can be alterations to placental growth, blood vessel development and function, and nutrient transportation. The changes to placental size and structure can be ascertained after delivery so serve as important biomarkers for changes in maternal gestational ecology. Fetal and childhood impacts of changes to maternal gestational ecology are varied and can include differences in growth as well as risk for anomalies such as congenital heart disease.

In many ways, modern health care accounts for cultural forces that subject humans to poor health [66]. Essential vitamins can be obtained in the form of manufactured supplements in lieu of being wholly dependent on dietary food provisions [58,67]. Respiratory illnesses, such as asthma, that can emerge from air pollution exposures are unideal but partially treatable [68,69]. Unfortunately, and despite advances in modern medicine, congenital heart disease continues to increase population mortality, but growing clinical focus on the role of the placenta in human disease may prove useful in future treatments [17,70].

## 8. Placental and Fetal Adaptation

The placenta is not a passive organ but can adapt in response to maternal or fetal signals [71]. For example, trophoblast thickness and capillary surface area, properties of a placenta, both exhibit continual changes as the fetus grows to decrease the distance between fetal and maternal circulations and increase surface area for nutrient exchange. Depending on the timing and nature of unfavorable or nutrient deficient conditions during fetal development, the placenta can also adapt to maintain normal growth and development. Marmoset monkey placentas have displayed plasticity to increase efficiency of resource allocations during triplet versus twin pregnancies [65]. While this adjustment does accompany costs to the triplet offspring, such as lower birthweight, prematurity, increased susceptibility to disease, and shorter lifespans, placental plasticity facilitates fetal survival [65]. Thus, life course processes, such as placental function during pregnancy, may exhibit tradeoffs that, while prioritizing survival, could lead to reduced development and increased disease susceptibility in offspring [61,65]. 

The placenta also relays information regarding the expecting parent’s environment, accounting for factors like malnutrition and hypoxia, to aid in fetal adaptation [72]. For example, deer mice living in hypoxic environments exhibit changes in the placenta that lead to reduced susceptibility to fetal growth restriction [73]. Famine and periods of scarcity can similarly affect both the placenta and fetus [74]. Depending on the trimester of pregnancy during which women experienced famine during the Dutch Hunger Winter of World War II, placentas were variably able to adapt their size and efficiencies to better support fetal growth. Famine during early stages of pregnancy lead to a lower placental–fetal weight ratio, indicating increased placental efficiency. By contrast, famine in later stages of pregnancy demonstrated reduced placental efficiency and a higher placental–fetal weight ratio. Maternal adiposity or body mass index can also impact fetal development, high body mass index is associated with lower levels of placental growth hormone and higher levels of inflammatory markers in Black women [75]. However, further research is needed to explain these relationships and to explore maternal social stresses and neonatal outcomes. Thus, the placenta aids in the fetus’ adaptability in the face of the changing maternal environment and resource availability, which can be considered in the context of social determinants of health [65].

## 9. Conclusions

A core tenet of the field of human evolutionary biology is to study how humans have adapted to survive and thrive in our environments [76]. This is especially relevant to the vulnerable periods of pregnancy, fetal development and early childhood. Considered one of the most rapidly evolving organs, placentas have emerged as warranted models at the intersection of human biology and evolution. The evolutionary perspective on placental biology highlights the unique structure and physiology of human placentas, and these facets of placental biology are strongly impacted by environmental and innate factors. Furthermore, studies investigating how the maternal environment may impact fetal growth, and outcomes could shed light on additional modes of placental plasticity. Congenital heart disease, which has multifactorial causes that involve gene–environment influences, is a worthwhile condition to explore such relationships. Given the strong evidence for placental biology as a potential mediator of risk and resiliency for neurodevelopmental impairment, modifications to placental dysfunction are an important future therapeutic aim. As the placenta continues to be a focus in both evolutionary biology and clinical spheres, the study of its developmental course and applications to long-term fetal health present a promising future for interventions.

## Data Availability

No new data were created or analyzed in this study.
